# Vaccine Candidate Against COVID-19 Based on Structurally Modified Plant Virus as an Adjuvant

**DOI:** 10.3389/fmicb.2022.845316

**Published:** 2022-02-28

**Authors:** Angelina O. Kovalenko, Ekaterina M. Ryabchevskaya, Ekaterina A. Evtushenko, Tatiana I. Manukhova, Olga A. Kondakova, Peter A. Ivanov, Marina V. Arkhipenko, Vladimir A. Gushchin, Nikolai A. Nikitin, Olga V. Karpova

**Affiliations:** ^1^Department of Virology, Faculty of Biology, Lomonosov Moscow State University, Moscow, Russia; ^2^N.F. Gamaleya National Research Center for Epidemiology and Microbiology, Ministry of Health of the Russian Federation, Moscow, Russia

**Keywords:** coronaviruses, vaccine candidate, plant virus, adjuvant, tobacco mosaic virus, structurally modified plant virus

## Abstract

A recombinant vaccine candidate has been developed based on the major coronaviruses’ antigen (S protein) fragments and a novel adjuvant—spherical particles (SPs) formed during tobacco mosaic virus thermal remodeling. The receptor-binding domain and the highly conserved antigenic fragments of the S2 protein subunit were chosen for the design of recombinant coronavirus antigens. The set of three antigens (Co1, CoF, and PE) was developed and used to create a vaccine candidate composed of antigens and SPs (SPs + 3AG). Recognition of SPs + 3AG compositions by commercially available antibodies against spike proteins of SARS-CoV and SARS-CoV-2 was confirmed. The immunogenicity testing of these compositions in a mouse model showed that SPs improved immune response to the CoF and PE antigens. Total IgG titers against both proteins were 9–16 times higher than those to SPs. Neutralizing activity against SARS-CoV-2 in serum samples collected from hamsters immunized with the SPs + 3AG was demonstrated.

## Introduction

The current COVID-19 pandemic, caused by severe acute respiratory syndrome coronavirus 2 (SARS-CoV-2), is the third outbreak of a dangerous coronavirus (CoV) infection of zoonotic origin in the human population in the twenty-first century. Like the pathogenic agents responsible for previous epidemics (SARS-CoV and MERS-CoV), the new SARS-CoV-2 virus belongs to the *Betacoronavirus* genus and can provoke severe acute respiratory syndrome, with a fatal outcome (Coronaviridae Study Group of the International Committee on Taxonomy of Viruses, 2020; Wu et al., 2020). The challenges of the SARS-CoV-2 pandemic underline the necessity to develop vaccines that will be resistant to viral antigen evolution. The design of a vaccine that will be effective against various SARS-CoV-2 strains, including emerging mutants, and other SARS-related coronaviruses is a current activity.

Spike protein (S protein) is the major antigen of coronaviruses that plays a key role in virions’ binding to receptors followed by penetration into the host cell. The S protein consists of two functional subunits: S1 attaches to the cell receptor through the receptor-binding domain (RBD) and S2 is responsible for the fusion between viral and host’s cell membranes (Huang et al., 2020). Mutations in S protein may lead to the interspecies barrier being overcome (Du et al., 2009a; Wan et al., 2020). Similar to other betacoronaviruses, the RBD of SARS-CoV-2 is the main target of neutralizing antibodies (nAbs) that block binding with the cellular surface and contribute the most to the neutralizing activity of convalescent patients’ sera (Piccoli et al., 2020; Robbiani et al., 2020; Steffen et al., 2020; Zost et al., 2020; Greaney et al., 2021a). The neutralizing activity of both monoclonal antibodies (mAbs) (Li et al., 2020; Greaney et al., 2021b) and polyclonal sera from SARS-recovered patients (Greaney et al., 2021a) is, however, considerably lower for emerging strains with mutations in RBD. Nevertheless, antibodies’ repertoire from different individuals possesses distinct antigen recognition ability and some serum samples neutralize viruses regardless of RBD mutations (Greaney et al., 2021a). Some human mAbs to the S2 subunit, in particular to epitopes in heptad repeat 1 (HR1) and in heptad repeat 2 (HR2), have been previously shown to provide broader protection against various SARS-CoV isolates (a causative agent of the 2002–2003 outbreak) than mAbs to the S1 subunit including RBD (Lip et al., 2006; Elshabrawy et al., 2012). Amino acid sequence analysis of SARS-CoV and SARS-CoV-2 has revealed highly conserved epitope sites in the S2 subunit (Ahmed et al., 2020). These data suggest that antibodies (Abs) against aforementioned conserved sites will induce a protective immune response to a wide range of coronaviruses (SARS-CoV, SARS-CoV-2, various SARS-related CoV of bats). Bat SARS-related coronaviruses are the focus of attention due to the potential danger of new epidemic outbreaks for humanity, since bats are natural reservoirs of coronaviruses (Menachery et al., 2015, 2016).

A reasonable solution for devising a vaccine with a good antigenic match to various coronaviruses is the application of conserved epitopes in recombinant antigen design, along with the use of approaches to efficient immune response induction. In the present study, genetically engineered constructs were created to provide expression of recombinant antigens in bacterial cells that included the RBD, as well as highly conserved (among various SARS-related CoV) fragments of the S2 subunit of the spike protein. The problem of individual proteins’ low immunogenicity was solved by the application of a novel safe adjuvant, spherical particles (SPs), generated by thermally induced structural remodeling of the tobacco mosaic virus (TMV). Previously, we have published pioneering works on the characterization of SPs’ properties, including adjuvant activity and safety (Atabekov et al., 2011; Nikitin et al., 2011, 2013, 2018b,2018c; Karpova et al., 2012; Trifonova et al., 2014, 2015; Ksenofontov et al., 2019; Evtushenko et al., 2020) and several vaccine candidates against pathogens of a viral and bacterial nature have been developed (Trifonova et al., 2017; Nikitin et al., 2018a; Kurashova et al., 2020; Ryabchevskaya et al., 2020, 2021). The authors assume that SPs in compositions with a target antigen act as a depot and this determines the adjuvant effect (Trifonova et al., 2017). Here, a vaccine candidate against COVID-19 representing a compositions of SPs and three recombinant antigens (SPs + 3AG) was obtained. A vaccine candidate for immunogenicity in mice and the ability to induce nAbs in hamsters was evaluated.

## Materials and Methods

### Construction of Coronavirus Antigens’ Expression Vectors

A pQE-Co1 expression plasmid contains a synthetic optimized RBD gene, which corresponds to 319–541 residues of SARS-CoV-2 S protein. It was assembled from overlapping oligonucleotides and cloned into a pQE-30 vector (Qiagen, Hilden, Germany) between *Bam*HI and *Sal*I restriction sites. To obtain a CoF synthetic gene, forward and reverse partially overlapping oligonucleotides, containing in their 5’-ends *Sal*I and *Hin*dIII restriction sites, respectively, and encoding selected conserved epitope from the HR2 region, were hybridized and elongated by the Klenow fragment (Fermentas, Vilnius, Lithuania). The elongated product was treated with appropriate enzymes and ligated with the pQE-Co1 vector. The resulting pQE-CoF plasmid mediated the expression of the fused protein. A synthetic optimized gene encoding PE protein was also assembled from overlapping oligonucleotides and ligated with the pQE-30 vector using *Bam*HI and *Hin*dIII sites that led to pQE-PE plasmid formation. pQE-Co1 and pQE-PE plasmids were assembled by the Evrogen Company (Moscow, Russia).

### Expression and Purification of Coronavirus Recombinant Antigens

*E. coli* strain SG13009 (Qiagen) was employed to express cloned genes. Bacterial cultures were grown overnight at 37°C in 6 ml of 2YT supplemented with kanamycin (final concentration 50 μg/ml) and ampicillin (final concentration 100 μg/ml). The cultures were transferred into 200 ml of fresh 2YT medium and incubated at 37°C with shaking at 180 rpm for 3 h. Then, an inducer (IPTG) was added to the final concentration of 2 mM and the biomass was grown for 4–5 h at 37°C. Cells were harvested by centrifugation at 5,000 rpm (JA-14 rotor, Avanti JXN-30 centrifuge, Beckman Coulter Inc., Brea, California, United States) for 10 min and pellets were resuspended in 5 ml of 6 M Guanidine Hydrochloride (GuHCl) lysis buffer supplemented with 0.2% sodium deoxycholate. Purification of (His)_6_-tagged proteins was carried out, according to the manufacturer’s protocol (Qiagen), by Ni-NTA chromatography under denaturing conditions. Proteins were dialyzed against Milli-Q (Simplicity UV, Merck Millipore, Darmstadt, Germany) in the ratio 1:250 for 4 h with hourly water changing. Proteins were sterilized using 0.2 μm filters (CHROMAFIL ^®^ CA-20/25(S), 729024). The theoretical molecular weights (Mr, kDa), isoelectric points (pI) and extinction coefficients (E_0_._1%_) of recombinant proteins were calculated using PROTPARAM from the Expert Protein Analysis System (EXPaSy) proteomics server^1^ of the Swiss Institute of Bioinformatics. Co1, CoF and PE concentrations were determined using spectrophotometry (U-2900 UV-VIS spectrophotometer, Hitachi, Japan) at 280 nm (E_280nm_ 0.1% Co1 = 1.233; E_280nm_ 0.1% CoF = 1.208). The PE antigen concentration was determined at 205 nm (E_205nm_ 0.1% = 31). Recombinant proteins were analyzed using SDS-PAGE (8–20% acrylamide linear gradient). Gels were stained with Coomassie brilliant blue G-250. Proteins’ molecular weights (Mr, kDa) were calculated using the ChemiDoc™ XRS + System with Image Lab™ Software (Bio-Rad Laboratories, Hercules, California, United States) by linear regression.

### Western Blot Analysis

The SDS-PAGE-separated proteins were transferred to an Amersham Hybond™-P polyvinylidene fluoride membrane (GE Healthcare, Chicago, Illinois, United States) by wet transfer. After that, the membrane was blocked with 5% non-fat dry milk in TBST (0.01 M Tris-HCl pH 7.4, 0.15 M NaCl, 0.05% Tween-20) and treated with rabbit polyclonal Abs to the SARS-CoV spike protein (Cat# MBS432054, MyBioSource, San Diego, California, United States) or rabbit polyclonal to the SARS-CoV-2 spike protein (Cat# MBS434243, MyBioSource) in a 1:1,000 dilution and then with secondary anti-rabbit Abs conjugated with horseradish peroxidase (W401B, Promega Corporation, Madison, Wisconsin, United States) in a 1:10,000 dilution. The WesternBright ECL HRP substrate (Advansta Inc., San Jose, California, United States) was used and the signal was detected by the ChemiDoc™ XRS + gel documentation system.

### Formation of Spherical Particles and Spherical Particles-Coronavirus Antigens Compositions

TMV was isolated and purified as previously described (Trifonova et al., 2015). SPs were obtained at a concentration of 2 mg/ml according to Trifonova et al. (2015). To obtain SPs-CoV antigens compositions (SPs + 3AG), 7 μg of each recombinant antigen (Co1, CoF and PE) was mixed with 250 μg of SPs in PBS (Cat# 70011-036, Gibco™, Thermo Fisher Scientific, Waltham, Massachusetts, United States).

### Transmission Electron Microscopy

The samples of SPs were obtained and analyzed as previously described (Evtushenko et al., 2020) by TEM microscope JEM-1400Flash (JEOL, Akishima, Tokyo, Japan). The scientific image manipulation software ImageJ (National Institutes of Health, United States) was used for size calculations.

### Endotoxin Levels of the Spherical Particles and Coronavirus Recombinant Antigens

Endotoxin levels of the SPs, each CoV recombinant antigen and Milli-Q water were determined using the endpoint chromogenic LAL assay with a minimum detection limit of 0.04 EU/ml. The LAL test and interpretation of results were performed according to the manufacturer’s protocol (Hycult Biotech Inc., Wayne, Pennsylvania, United States). Briefly, a standard endotoxin stock solution with a concentration of 50 EU/ml was prepared and used in serial dilutions to construct endotoxin standard curve. 50 μl of LAL reagent was added to 50 μl of standard serial dilutions or samples in endotoxin free 96-well microplates. After 20 min of incubation at 25°C the enzymatic reaction was stopped by the addition of 50 μl of 20% acetic acid. The absorbance at 405 nm was measured. The concentration of endotoxin in the samples was determined using the endotoxin standard curve. Endotoxin level of the Milli-Q water was 0.049 EU/ml.

### Immunofluorescence Microscopy

Immunofluorescence microscopy of compositions was performed as previously described (Ryabchevskaya et al., 2020). SPs + 3AG compositions were treated with primary rabbit polyclonal Abs to the SARS-CoV spike protein (MBS432054) or rabbit polyclonal Abs to the SARS-CoV-2 spike protein (MBS434243) in a dilution of 1:50, and secondary Abs conjugated to Alexa Fluor ^®^ 546 (Invitrogen™, ThermoFisher Scientific, United States) in a dilution of 1:500. The results of immunostaining were analyzed using an Axiovert 200M fluorescence microscope (Carl Zeiss, Göttingen, Germany) equipped with an ORCAII-ERG2 integrated camera (Hamamatsu Photonics K.K, Hamamatsu City, Japan).

### Immunization of Mice and Hamsters

White outbred female mice (age 6–8 weeks; weight 20–25 grams) were used. On the proof-of-concept stage, two experimental groups were selected to study the immunogenicity of the vaccine candidate. Each experimental group contained ten animals. The negative control (group 1) consisted of five animals immunized with PBS. All groups of mice were immunized with two intraperitoneal injections at 2-week intervals. The scheme of the experiment to study the immunogenicity of the vaccine candidate is presented in the “Results” section (paragraph 4 « SPs enhance immune response to the CoV antigens »). For group 2, one dose of the formulation of the individual CoV-antigens mixture contained 7 μg of Co1 antigen, 7 μg of CoF antigen and 7 μg of PE antigen. For group 3, one dose of the SPs + 3AG compositions contained 7 μg of Co1 antigen, 7 μg of CoF antigen, 7 μg of PE antigen and 250 μg of SPs. All samples administered were in PBS up to the final volume of 0.2 ml. Using mice was approved by the ethics committee of the Lomonosov Moscow State University (Application # 136-a, version 3, approved during the Bioethics Commission meeting # 134-d held on 19.08.2021).

A group of ten female Syrian hamsters (age 8–10 weeks; weight 64–106 grams) was immunized twice with the vaccine candidate (SPs + 3AG compositions) at 3-week intervals by the intramuscular route. The dose (μg) of SPs + 3AG compositions was the same as for the mice. The volume of injection was 0.5 ml. Fractional injections in two pelvic limbs, in equal amounts (0.25 ml), with a 1-h interval, were used due to the need to limit the maximum possible volume for intramuscular immunization. Blood was collected 21 days after the second immunization, from the gingival vein, in order to evaluate sera neutralizing activity. The study on hamsters was approved by the Commission on Bioethics (BEC) of a Group of scientific research institutes (protocol #9.73/20 dated 28.12.2020).

### Indirect Enzyme-Linked Immunosorbent Assay

ELISA was performed as previously described (Evtushenko et al., 2020) with some modifications. 96-well microplates (Greiner Bio-One 655001, Germany) were coated with 10 μg/mL of Co1/CoF/PE/SPs and incubated overnight at 4°C. Then, microplates were washed five times with a PBS containing 0.1% Tween-20 (PBST) and blocked for 2 h at room temperature with a PBS containing 1% skimmed milk. Sera from mice were titrated in serial dilutions with PBS containing 1% non-fat dry milk in 3-fold steps. Microplates were incubated overnight at 4°C and then washed five times with PBST. Anti-mouse HRP conjugates (Abcam, Cambridge, United Kingdom) against IgG (ab6728), IgG1 (ab97240), IgG2a (ab97245), IgG2b (ab97250), and IgG3 (ab97260) were used in a dilution of 1/10,000. After incubation for 1 h at 37°C, the microplates were washed five times with PBST. After TMB-staining and A_450_ measuring, a positive titer was defined as three standard deviations above the mean value of the block. The median titers were calculated for each group.

### Cells and SARS-CoV-2 Strain

Vero E6 cells (ATCC, CRL-1586) were maintained in complete DMEM medium (PanEco, Russia), supplemented with 10% FBS (fetal bovine serum; HyClone, United States), 1 × penicillin-streptomycin (250 U/ml and 250 μg/ml; PanEco, Russia) at 37°C and 5% CO_2_. The SARS-CoV-2 strain hCoV-19/Russia/Moscow_PMVL-4/2020 (GISAID ID EPI_ISL_470898) was isolated from the nasopharyngeal aspirate specimen of a patient with COVID-19. All experiments with live SARS-CoV-2 were performed at a biosafety level-3 (BSL-3) facility.

### Virus Neutralization Assay

Evaluation of the hamsters’ sera neutralizing activity was performed on the 21st day after the second immunization. Serum-neutralizing titers were determined as previously described (Gushchin et al., 2021) with some modifications, using the SARS-CoV-2 strain PMVL-4. Before the neutralization test, hamsters’ sera were incubated at 56°C for 1 h. Sera dilutions were prepared in complete DMEM growth medium. Sera dilutions were mixed with 100 TCID_50_ of SARS-CoV-2 and incubated at 37°C for 1 h. Then, serum-virus mixture was added to a 96-well plate with a Vero E6 cell monolayer and incubated in 5% CO_2_ at 37°C for 96 h. The virus-induced cytopathic effect (CPE) was assessed using the MTT assay (Siniavin et al., 2021). The half maximal neutralizing titer (NT_50_) for sera was determined using non-linear regression, i.e., log (inhibitor) vs. normalized response—Variable slope model, using GraphPadPrism version 9.1.0 (GraphPad Software Inc., San Diego, California, United States).

### Statistical Analysis

For multiple comparisons, the Kruskal-Wallis test and a *post hoc* Dunn’s test were used. The Wilcoxon-Mann-Whitney Test was used to compare differences between two groups. The statistical tests used in each individual experiment have been indicated in figure captions. Probability values (*P*-values) of less than 0.05 were considered to be significant. The statistical processing of the results was carried out using GraphPadPrism 9.1.0.

## Results

### Coronavirus Recombinant Antigens Design

**FIGURE 1:**
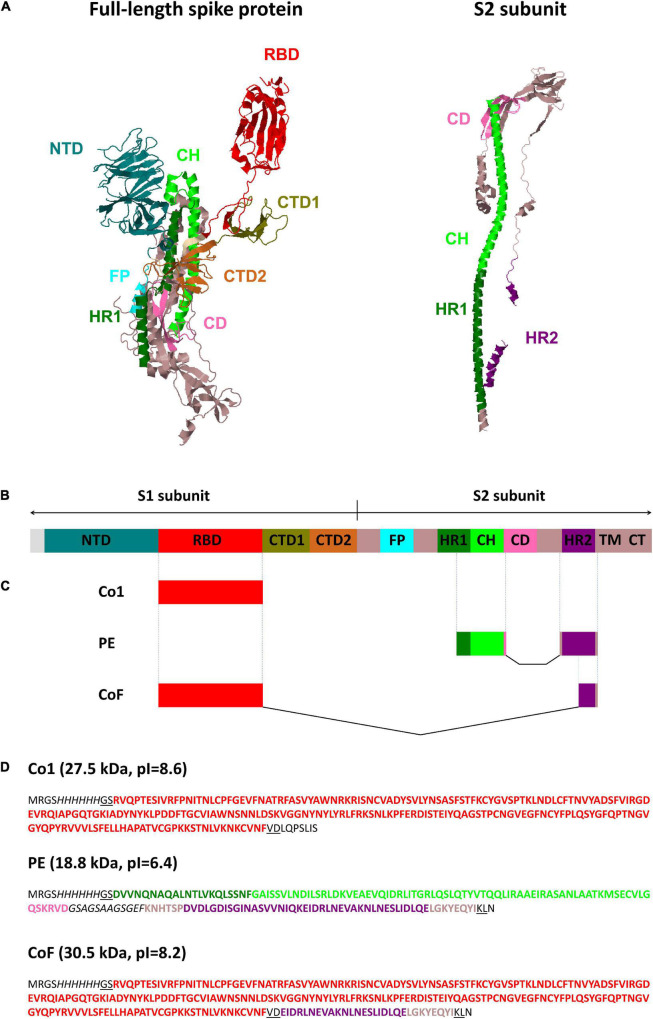
SARS-CoV-2 spike protein structure and graphical overview of CoV recombinant antigens Co1, PE and CoF. **(A)** Structural model of the full-length SARS-CoV-2 spike protein (PDB code: 6VSB) (Berman et al., 2000; Wrapp et al., 2020) and S2 subunit (PDB code: 6XRA) (Berman et al., 2000; Cai et al., 2020). Protein structures were visualized using Jmol software (http://www.jmol.org). **(B)** Linear diagram of the full-length SARS-CoV-2 spike protein domain structure in the same color scheme as **(A)** (not to scale). NTD, N-terminal domain; RBD, receptor-binding domain; CTD1, CTD2, C-terminal domain 1 and 2; FP, fusion protein; HR1, HR2, heptad repeat 1 and 2; CH, central helix; CD, connector domain; TM, transmembrane domain; CT, cytoplasmic tail. **(C,D)** Graphical color schemes (not to scale) and amino acid sequences of CoV recombinant antigens Co1, PE, and CoF. Coronaviruses’ amino acid sequences are highlighted in bold and colored corresponding to the **(A–C)**; glycine-serine linker within PE protein and (His)_6_-tags are italicized; amino acid residues encoded by restriction sites used for cloning (*Bam*HI, *Hin*dIII, *Sal*I) are underlined; sequences encoded by the pQE30 vector, including the polylinker fragments, are shown with normal font. Domains are colored according to the coordinates indicated in Sun et al. (2021).

Spike protein (S protein) is the major coronavirus antigen. The S protein is cleaved into S1 (aa 13–685) and S2 (aa 686–1,273) subunits by cellular proteases. The S1 subunit binds to the cell receptor and is subdivided into four domains: N-terminal domain (NTD) (aa 13–306), receptor-binding domain (RBD) (aa 319–541), C-terminal domain 1 (CTD1) and C-terminal domain 2 (CTD2). The S2 subunit mediates membrane fusion and contains fusion peptide (FP), heptad repeat 1 (HR1) (aa 920–970), central helix (CH) (aa 971–1035), connector domain (CD), heptad repeat 2 (HR2) (aa 1,163–1,202), transmembrane domain (TM) and C-terminal domain (CT) (Sun et al., 2021; Figures 1A,B).

At the first stage of the present study, the RBD of S protein was selected for inclusion in a vaccine candidate. The antigen consisting of the sequence corresponding to the SARS-CoV-2 RBD (aa 319–541) was named Co1 (Figures 1C,D). The RBD amino acid residues are known to be variable, therefore the vaccine candidate must contain conserved epitopes to provide a protective immune response against both SARS-CoV-2 and SARS-CoV. Such epitopes were identified within sequences of the S2 subunit. A selection was made of conserved S2 subunit fragments 950–1,041 (including part of HR1 and CH domains) and 1,157–1,210 (including the HR2 domain), which coincide (up to 100% homology) with sequences of human SARS-CoV (GenBank AAS10463, ADC35483) and SARS-CoV-2 (Wuhan-Hu-1, NC_045512). This statement is also true for some bat species’ coronaviruses (for *Rhinolophus affinis*—QDF43830, *Rhinolophus sinicus*—AIA62320, *Rhinolophus ferrumequinum*—ATO98145, *Chaerephon plicata*—AGC74176) including coronavirus RaTG13 (QHR63300), coronaviruses of palm civets (AAV97990, AAU04664), pangolins (QIA48641) and SARS-CoV after passages on ferrets (AFR58714) or Vero E6 cells (BAE93401). Both sites contained previously predicted T-cell and B-cell epitopes that partially overlap with each other (Ahmed et al., 2020; Grifoni et al., 2020). On the base of these conserved S2 subunit sites, a recombinant polyepitope antigen named PE (Figures 1C,D) was obtained. The connection of functional domains without a linker can lead to improper folding of the protein and reduce its bioactivity. Typically, the design of such a linker assumes the presence of flexible and hydrophilic residues (Chen et al., 2013). The PE antigen, in addition to selected antigenic regions, contained the glycine/serine-rich sequence **GSAGSAAGSGEF** (Waldo et al., 1999) which does not include common repetitive motifs, for instance (G_3_S), (G_4_S) or (G_3_A). In addition to Co1 and PE antigens, it was decided to supplement the Co1 antigen (containing RBD) with one of conserved epitopes from the S2 subunit. According to previous studies, Abs specific to the sequence 1,157–1,210 including the epitope **EIDRLNEVAKNLNESLIDLQELGKYEQYI** [residues 1,182-1,210 in the coordinates of the reference sequence (Wuhan-Hu-1, GenBank YP_009724390.1)] were isolated from the blood sera of humans with SARS pneumonia. These Abs were demonstrated to be able to bind to SARS-CoV virions, as well as to block the infection in Vero E6 cells and in experiments on monkeys (Wang et al., 2016). The protein containing RBD fused to the 29 amino acid residues of the HR2 domain was named CoF (Figures 1C,D).

The design of all genetically engineered constructs was based on the pQE-30 vector. The gene encoding RBD was cloned between *Bam*HI and *Sal*I restriction sites to obtain the pQE-Co1 plasmid. Since *Sal*I does not occupy the 3’-terminal position within the pQE-30 multiple cloning sites (MCS) region, there was an option to incorporate any sequence at the 3’-end of the RBD-coding sequence using one-step cloning between *Sal*I and *Hin*dIII restriction sites. Thus, the design of the pQE-Co1 plasmid assumed the possibility of creating proteins fused with the C-terminal region of RBD. Because of this, the pQE-CoF plasmid for the expression of fusion protein consisting of the RBD domain and the conserved epitope (aa 1,182–1,210) was constructed.

The sequences of all CoV recombinant antigens were subjected to “reverse translation” *in silico*, taking into account the frequency of codon use in *Escherichia coli*. Optimization procedures of the nucleotide sequences obtained provided for increased variability of triplets encoding the same amino acid (in the case of comparable codon frequencies in *E. coli*), “mutagenesis” of various cis-acting elements that negatively affect the efficiency of expression in bacterial cells (cryptic transcription promoters and Shine-Dalgarno sequences, A/T-rich regions, direct repeats), as well as, if necessary, emerging restriction sites used during cloning (Ryabchevskaya et al., 2021).

### Expression and Analysis of Coronavirus Antigens

**FIGURE 2:**
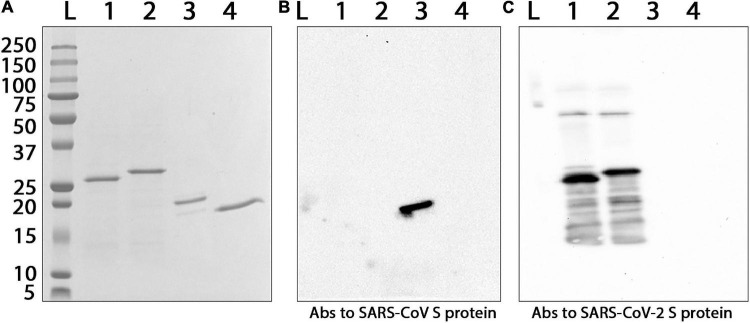
Interaction of the purified CoV recombinant antigens with commercial polyclonal Abs against S proteins of different coronaviruses. **(A)** Electrophoretic analysis in 8–20% SDS-PAGE, staining by Coomassie G-250. **(B)** Western blot analysis with primary polyclonal anti-spike (SARS-CoV) Abs. **(C)** Western blot analysis with primary polyclonal anti-spike (SARS-CoV-2) Abs. Secondary HRP-conjugated Abs were used. L, protein molecular weight markers ladder (molecular weights in kDa are indicated on the left); Abs, antibodies; 1, Co1; 2, CoF; 3, PE; 4, TMV coat protein (negative control).

The recombinant proteins were purified by metal affinity chromatography. The yield of Co1, CoF and PE was 8, 30, and 120 mg, respectively, per 1 L of the culture medium. Endotoxin levels of each CoV recombinant antigen were measured using the endpoint chromogenic LAL assay. The endotoxin content was 5.48 EU/mg for Co1 antigen, 1.24 EU/mg for CoF antigen and 23.15 EU/mg for PE antigen. The molecular weights of the recombinant proteins (Mr, kDa) determined by electrophoretic analysis (Figure 2A) were consistent with the values calculated using the PROTPARAM tool, which were 26.5 kDa for Co1 (theoretical value 27.5 kDa), 28.2 kDa for CoF (theoretical value 30.5 kDa) and 18.1 kDa for PE (theoretical value 18.8 kDa). The antigenic properties of the recombinant proteins were confirmed by Western blot analysis (Figure 2). PE antigen interacted with commercial polyclonal anti-spike (SARS-CoV) Abs (Figure 2B, lane 3). Co1 and CoF antigens reacted with polyclonal anti-spike (SARS-CoV-2) Abs (Figure 2C, lanes 1,2, respectively).

### Spherical Particles Compositions With Coronavirus Antigens

**FIGURE 3:**
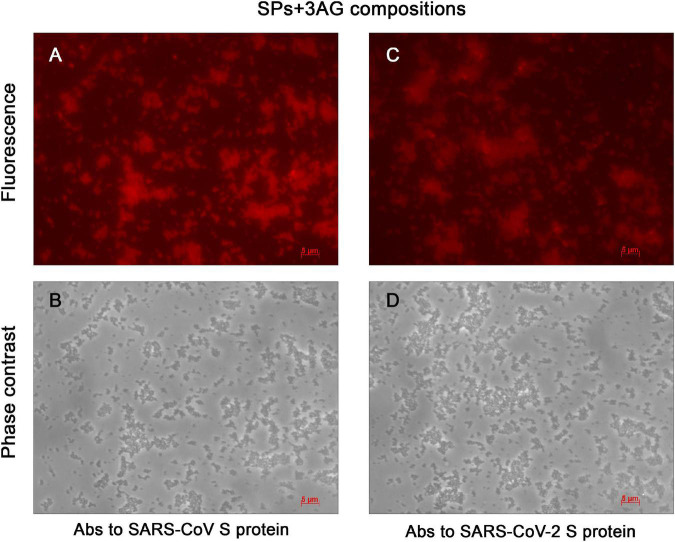
Immunofluorescent analysis of SPs + 3AG compositions. **(A)** and **(B)**, **(C)** and **(D)** In pairs are the same images presented in fluorescence and phase contrast modes, respectively. The SPs + 3AG compositions were treated with polyclonal anti-spike (SARS-CoV) Abs **(A,B)** or polyclonal anti-spike (SARS-CoV-2) Abs **(C,D)** and secondary Abs conjugated to Alexa Fluor ^®^ 546. Scale bars, 5 μm. Complexes were obtained in 1 x PBS. Abs, antibodies.

SPs were characterized using transmission electron microscopy (TEM) (Supplementary Figure 1). The mean diameter of SPs, measured with ImageJ software (NIH, United States), was 466 ± 83 (mean ± *SD*, *n* = 100). Endotoxin level of the SPs was measured using the endpoint chromogenic LAL assay. The endotoxin content was 0.073 EU/mg. In order to produce the vaccine candidate, three recombinant antigens (AG): Co1, CoF, and PE, were mixed with SPs, which were plant-based adjuvant previously developed in the authors’ laboratory (Karpova et al., 2012; Trifonova et al., 2017; Evtushenko et al., 2020). Compositions consisted of 7 μg of each recombinant antigen and 250 μg of SPs (SPs + 3AG) and were obtained in PBS, which is an acceptable buffer for vaccine formulations. Total antigen mass amounted to 21 μg and the SPs/antigens mass ratio was 250/21, which is close to the ratio of 10/1 that was previously shown to be appropriate for effective immunostimulation (Trifonova et al., 2017). SPs + 3AG compositions were analyzed by indirect immunofluorescent microscopy, with polyclonal Abs to full-size S protein SARS-CoV (Figures 3A,B) or SARS-CoV-2 (Figures 3C,D) as primary Abs and Alexa Fluor ^®^ 546-conjugated secondary Abs. The presence of the red fluorescent signal corresponding to the location of SPs indicated that antigens adsorbed to the SPs kept their antigenic specificity within the compositions formed. Comparison of images obtained in fluorescent (Figures 3A,C) and phase contrast (Figures 3B,D) modes reveals that all SPs were covered by molecules of antigens. The clear recognition by both Abs of the SARS-CoV S protein (Figure 3A) and the SARS-CoV-2 S protein (Figure 3C) confirms that epitopes corresponding to both coronaviruses are available for interaction within SPs + 3AG compositions. This suggests that these compositions are able to induce an immune response against both SARS-CoV and SARS-CoV-2. The result of immunofluorescence analysis of SPs without 3AG (negative controls) are represented in Supplementary Figure 2.

### Spherical Particles Enhance Immune Response to the Coronavirus Antigens

**FIGURE 4:**
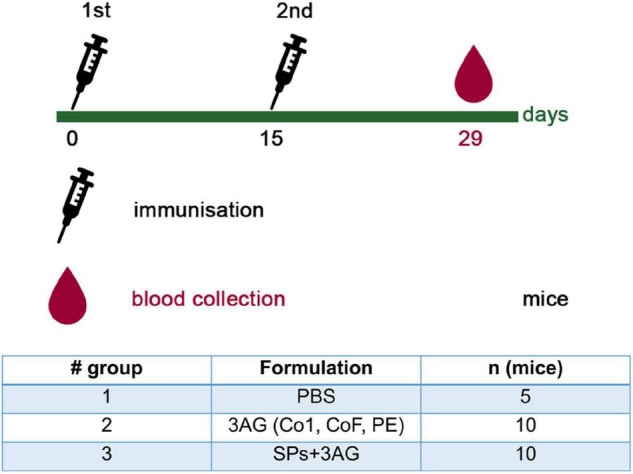
Immunization schedule and description of animal groups involved in the experiment to evaluate CoV antigens’ immunogenicity. Groups of mice were injected intraperitoneally either with 21 μg of CoV antigens (7 μg of each antigen) (group 2) or with 21 μg of CoV antigens in compositions with SPs (250 μg) (group 3). The control group was injected with PBS. All samples were administered with PBS in a total volume 0.2 ml. AG, antigen; SPs, spherical particles obtained by the thermal remodeling of TMV; n, number of animals.

**FIGURE 5:**
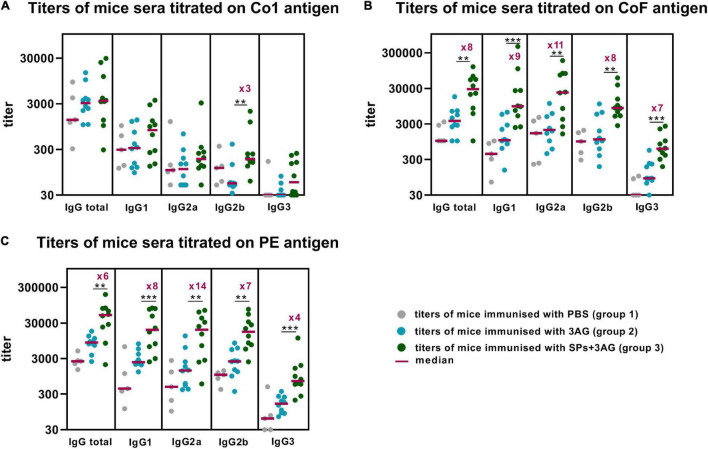
Enhancement of immune response to CoV antigens by SPs. Groups of mice were immunized intraperitoneally twice (days 0, 15). Blood was collected after the second immunization on the 29th day of the experiment. Sera titers were evaluated using an indirect ELISA. Antigen concentrations on microplate—10 μg/ml for all antigens. *P-*values were calculated using a Mann-Whitney *U*-test. **P < 0.01; ***P < 0.001. **(A)** IgG titers to Co1. **(B)** IgG titers to CoF. **(C)** IgG titers to PE.

To evaluate the ability of SPs + 3AG compositions to induce antibody production against CoV antigens mice were immunized twice intraperitoneally, with a 2-week interval. A group of mice was injected with SPs + 3AG compositions which consisted of 21 μg of CoV antigens (7 μg of each antigen) and 250 μg of SPs in 0.2 ml of PBS solution (group 3). The other group was injected with 0.2 ml of PBS solution containing the same dose (21 μg) of CoV antigens (7 μg of each antigen) in the absence of SPs (group 2). Endotoxin level of the 3AG formulation (7 μg of Co1 antigen, 7 μg of CoF antigen, 7 μg of PE antigen) was 0.217 EU/dose and endotoxin level of the vaccine candidate formulation (7 μg of Co1 antigen, 7 μg of CoF antigen, 7 μg of PE antigen and 250 μg of SPs) was 0.229 EU/dose. As a control (group 1), a group of mice immunized with PBS (0.2 ml) was used. We did not immunize mice with SPs alone because it was not relevant for this study. However, in our previously studies we have shown that after administration of SPs alone, antibodies to candidate vaccine antigen were absent in sera of immunized animals (Kurashova et al., 2020). The immunization schedule and the groups’ description summary are represented in Figure 4. The general condition of the animals during the immunization period was monitored. There were no animal deaths and no signs of abnormalities. Two weeks after the second immunization, blood was collected and the levels of anti-Co1 (Figure 5A), anti-CoF (Figure 5B) and anti-PE (Figure 5C) total IgG, as well as IgG1, IgG2a, IgG2b, and IgG3 subclasses, were measured by indirect ELISA.

Comparison of the sera titers collected from the mice belonging to groups 2 and 3, which differed only in the presence of SPs, made it possible to establish SPs’ influence on CoV antigens’ immunogenicity within SPs + 3AG compositions. SPs were shown to significantly enhance the immune response to the CoF antigen (median total IgG titer–28505) (Figure 5B and Supplementary Table 2) and to the PE antigen (median total IgG titer–49,805) (Figure 5C and Supplementary Table 1), compared to the anti-CoF (median total IgG titer–3,645) titers and to the anti-PE (median total IgG titer—8,511) elicited by the mixture of individual antigens (group 2). A study of IgG subclasses revealed that not only total IgG titers, but also IgG1, IgG2a, IgG2b, and IgG3 titers of Abs specific to both CoF and PE antigens, were significantly higher in sera samples from the group immunized with SPs + 3AG compositions (group 3) than from the one immunized with the mixture of individual antigens (group 2) (Figures 5B,C and Supplementary Tables 1, 2). As far as the Co1 antigen is concerned, no difference in anti-Co1 total IgG titers in serum samples collected from the animals of groups 3 and 2 was registered. A statistical significance was revealed for IgG2b titers which were three times higher after immunization with SPs + 3AG compositions (group 3) (median IgG2b titer–185) than after immunization with the mixture of CoV antigens without SPs (group 2) (median IgG2b titer–55) (Figure 5A). However, it also should be noted that there were no statistically significant differences between the group immunized with PBS (group 1) (median IgG2b titer–118) and group 3 in Dunn’s multiple comparison test (Supplementary Table 3).

### Comparative Analysis of the Immune Response to Spherical Particles and Coronavirus Antigens

**FIGURE 6:**
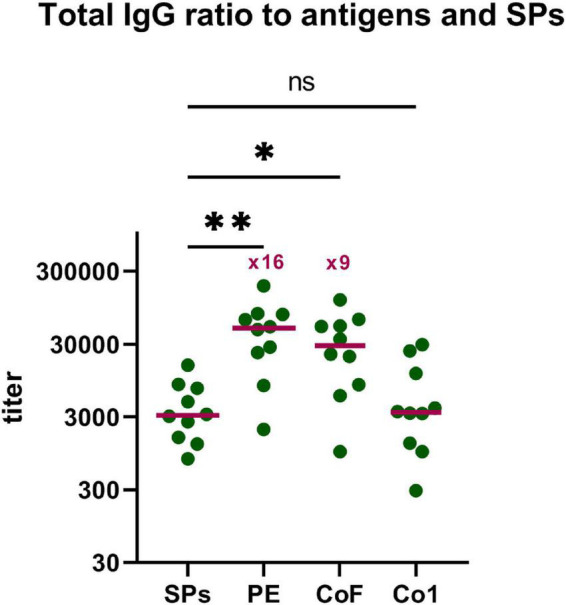
Comparative analysis of the immune response to SPs and CoV antigens. Group of mice (group 3) was immunized intraperitoneally twice (days 0, 15) with SPs + 3AG compositions. Blood was collected after the second immunization on the 29th day of the experiment. Sera titers were evaluated using an indirect ELISA. Concentration of SPs and CoV antigens on microplate—10 μg/ml. •, titers of mice; –, median. *P-*values were calculated using a *post hoc* Dunn’s multiple-comparison test, which was conducted after a Kruskal-Wallis test. Kruskal-Wallis test *P-*value: 0.0012. *P < 0.05; ^**^P < 0.01; ns, not significant.

The other aspect of practical importance is the ratio of sera titers to the SPs and to the CoV antigens after immunization by SPs + 3AG compositions. To find the ratio of Abs produced to the SPs and to the target antigens (Co1, CoF, PE), total IgG titers to SPs in mice sera obtained after the second immunization with SPs + 3AG compositions were analyzed in the same way as anti-CoV antigens (paragraph 4 « SPs enhance immune response to the CoV antigens » of the « Results » section). A comparison of total IgG titers specific to the SPs and to each of the CoV antigens is presented in Figure 6. The titer (median) of total IgG to the Co1 antigen was low and did not differ from that relating to SPs (Figure 6 and Supplementary Tables 3, 4). Meanwhile, two immunizations of mice with SPs + 3AG compositions were shown to lead to the induction of 9-fold, and 16-fold, higher total IgG titers to the CoF and the PE, respectively, than to the SPs serving as adjuvant (Figure 6 and Supplementary Tables 1, 2, 4).

### Protective Efficacy Against Live SARS-CoV-2 in Cell Culture

**FIGURE 7:**
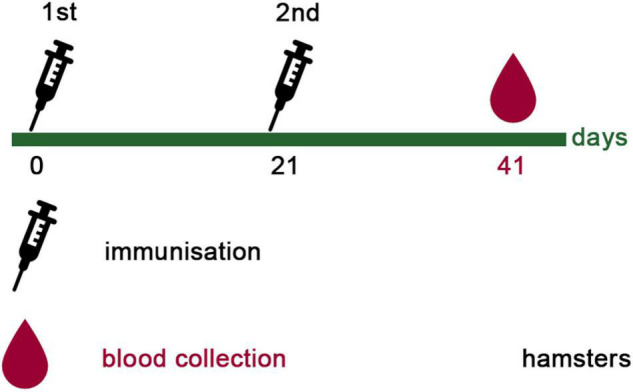
Immunization schedule of hamsters. Hamsters (ten females) were immunized intramuscularly with 21 μg of CoV antigens in compositions with SPs (250 μg) twice, with a 3-week interval. All samples were administered with PBS in a total volume 0.5 ml. Twenty-one days after the second immunization, blood was collected in order to evaluate sera neutralizing activity.

**FIGURE 8:**
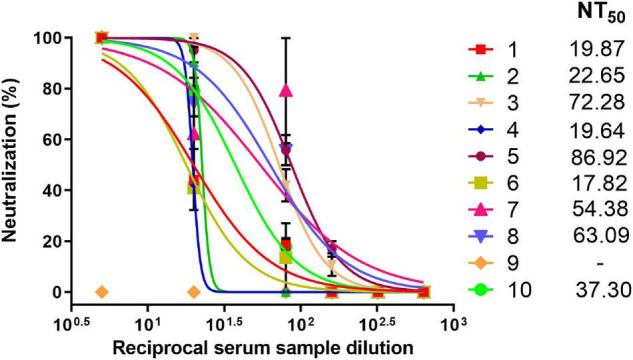
Neutralizing activity of hamster sera against SARS-CoV-2. Dose-response curves of sera neutralizing activity against live SARS-CoV-2. Data represent mean values ± SEM of n = 3 technical replicates. The numbers (1–10) indicate individual sera. NT_50_–viral neutralization activity with 50% neutralization.

Since the SPs + 3AG compositions were shown to provide better immune response than 3AG alone in mice, at the current stage we decided to evaluate the sera neutralizing activity of animals immunized only with the vaccine candidate (SPs + 3AG). For this purpose, Syrian hamsters were immunized intramuscularly with SPs + 3AG compositions (7 μg of each recombinant antigen and 250 μg of SPs in PBS per hamster). Endotoxin level of the vaccine candidate formulation was 0.242 EU/dose. A second equivalent dose was administered 21 days later. During the period of immunization, animals were monitored daily and body weight was controlled (on days 0, 21, and 42). No mortality of hamsters and nor any observed signs of deviations were detected. The considerable weight loss of animals was not observed (Supplementary Table 5). Serum samples were collected from the hamsters 21 days after the second immunization and tested for neutralizing activity. The schedule of immunization is shown in Figure 7. Neutralizing activity (NT_50_) was determined using live SARS-CoV-2 on the Vero E6 cell. Immunized hamsters’ serum samples were shown to display neutralizing activity with NT_50_ values from 17.82 to 86.92 (Figure 8). This is evidence that SPs + 3AG compositions were able to induce virus-neutralizing antibodies against SARS-CoV-2 in hamsters and therefore can be considered to be a promising coronavirus vaccine candidate.

## Discussion

Vaccines against SARS-CoV-2 (both under development and licensed) are based on different approaches: (1) whole-virion vaccines (inactivated and live-attenuated); (2) nucleic acid vaccines that provide an *in vivo* expression of target recombinant antigens in host cells (viral vector vaccines, DNA vaccines, RNA vaccines); (3) vaccines that comprise recombinant proteins and virus-like particles produced in various expression systems. According to WHO (07.09.2021), in September 2021, 299 vaccines were at different stages of preclinical studies and clinical trials. More than 89% of these vaccines contained recombinant antigens.^2^ Full-length S protein and RBD are the most commonly used amino acid sequences for recombinant COVID-19 vaccine candidates (see text footnote 2) (Corbett et al., 2020; Folegatti et al., 2020; Keech et al., 2020; Kuo et al., 2020; Logunov et al., 2020; Yang et al., 2020; Zhu et al., 2020; Chen et al., 2021; Lamb, 2021; Lee et al., 2021; Liang et al., 2021; Sadoff et al., 2021). Some vaccines based on the whole S protein were introduced urgently during mass vaccination at the time of the pandemic. The potential risks and concerns associated with such vaccines should, however, not be underestimated. Therefore, it remains desirable to develop new vaccines based on other platforms, due to the obvious necessity for revaccination.

Previously, it was shown that Abs specific to the certain sites of SARS-CoV S protein can induce hypercytokinemia and aggressive inflammation, leading to acute lung injury (ALI) syndrome (Liu et al., 2019), as well as antibody-dependent enhancement (ADE) of viral infection. Inactivated vaccines against SARS-CoV, MERS-CoV and recombinant vaccines based on SARS-CoV full-length S protein induced ADE and caused lung pathology, and this stopped their further application (Jaume et al., 2012; Tseng et al., 2012; Agrawal et al., 2016; Wang et al., 2016). Although the risk of ADE triggered by SARS-CoV-2 vaccines has not been shown, this question continues to be subject to close scrutiny (Arvin et al., 2020; Lee et al., 2020; Cardozo and Veazey, 2021; Ricke, 2021). Abs against S597-603 epitope, that is identical for SARS-CoV and SARS-CoV-2, could activate ADE in the case of SARS-CoV (Wang et al., 2016). Consequently, there is a need to create new recombinant vaccines that will include the most promising antigenic determinants and will not contain epitopes associated with the potential complications of ADE.

The set of SARS-CoV-2 recombinant antigens for vaccine candidate containing structurally modified plant virus was developed during the early stages of the current pandemic. SARS-CoV-2 RBD is a part of the S glycoprotein surface that provides interaction with the cellular receptor ACE2 (angiotensin-converting enzyme 2) (Wrapp et al., 2020). The similarly well-studied domain of the related coronavirus SARS-CoV binds to the same receptor (Li et al., 2003). Peptide mapping and subsequent computer analysis confirmed the high immunogenic potential of RBD (Tarnovitski et al., 2006). Several monoclonal Abs specific to linear and conformational RBD epitopes were isolated from mice immunized with the inactivated SARS-CoV vaccine. Some of these Abs blocked the interaction between RBD and ACE2, but neutralization effectiveness depended on various point mutations (He et al., 2006).

Considering previously published SARS-CoV research (Lip et al., 2006; Elshabrawy et al., 2012), in the present study evolutionarily conserved sites of S2 subunit were selected in addition to the RBD. Three recombinant antigens (3AG: Co1, CoF, and PE) were constructed and expressed. The Co1 protein contained the sequence of the RBD of the SARS-CoV-2 S protein. The CoF protein was composed of SARS-CoV-2 RBD fused to the highly conserved epitope from the HR2 of the S2 subunit. Polyepitope protein (PE) was derived from conserved S2 subunit fragments containing the heptad repeats HR1 (C-terminal fragment), HR2 and CH domain (Figure 1). The selected conserved amino acid sequences from the S2 subunit are similar (up to 100% homology) in various coronaviruses: SARS-CoV, SARS-CoV-2, coronaviruses of four bat species (*Rhinolophus affinis*, *Rhinolophus sinicus*, *Rhinolophus ferrumequinum*, and *Chaerephon plicata*) including coronavirus RaTG13 as well as coronaviruses of palm civets (*Paguma larvata)* and pangolins (*Manis javanica*). This suggests that Abs to selected highly conserved sites will provide a protective effect against emerging mutants of SARS-CoV-2 and various SARS-related CoV, in particular some bat viruses, which could be a source of new coronavirus outbreaks. The Co1 and CoF constructs have a flexible “modular” design that enables upgrade by fusion with different target epitopes. Thus, the developed antigen set could be adapted and modified for new betacoronavirus threats.

Co1, CoF and PE have been obtained in *E. coli*. This expression system has been used for over 40 years and has well-known advantages (Rosano and Ceccarelli, 2014). A drawback of this expression system is the lack of post-translational modification of proteins, in particular protein glycosylation, which is common for eukaryotic cells. However, the *E. coli* expression system is applied for glycoprotein production if glycans’ absence does not affect protein function (Kamionka, 2011). Previously, recombinant proteins based on SARS-CoV RBD, which, by analogy with SARS-CoV-2 RBD, has N-glycosylation sites in the N-terminal region, have been obtained in various expression systems, including mammalian cells, insect cells and *E. coli.* The immunogenicity and protective efficacy of proteins were compared, and it was found that all proteins induced a potent nAbs response and protected mice against SARS-CoV. These data indicated that any of the tested expression systems were suitable for RBD-based antigens production (Du et al., 2009b). In contrast to Co1, which comprises RBD, PE antigen and the conserved epitope within the CoF protein did not contain N-glycosylation sites. Recently, SARS-CoV-2 RBDs produced in *E. coli* and in mammalian HEK293 cells were compared (He et al., 2021). Proteins’ structure was characterized and the ability of both proteins to bind with the ACE2 receptor was assessed. The authors detected no substantial effects of different expression systems on the secondary and tertiary structure of proteins and receptor binding. Thus, RBD obtained in *E. coli* can be applied for vaccines’ and test systems’ development. At the same time, the protein yield in *E. coli* was 13.3 mg/L of the cell culture and only 5 mg/L in mammalian cells (He et al., 2021). In the current work, the yield of purified Co1, containing RBD, was 8 mg/L. The yield of CoF, constituted RBD and fused conserved S2 epitope was 30 mg/L. PE antigen had the highest yield: 120 mg/L.

At the first stage of the study, the possibility of recombinant proteins interacting with commercially available polyclonal Abs was examined using Western blot analysis. The Co1 and CoF antigens reacted with Abs to the full-size S protein of SARS-CoV-2 but did not bind with Abs to the full-size S protein of SARS-CoV. A sequence comparison has shown that the SARS-CoV RBD has only 75% homology with the SARS-CoV-2 RBD at the level of the amino acid sequence (Huang et al., 2020). Previously, it was demonstrated that polyclonal Abs from convalescent sera to SARS-CoV provide a limited level of cross-interaction with SARS-CoV-2 lentivirus-based pseudovirions and are unable to prevent SARS-CoV-2 interaction with the host cell receptor (Ou et al., 2020). Although CoF was recognized only by commercial Abs to SARS-CoV-2, it was also included into the vaccine candidate because of its high yield and conserved HR2 epitope, which had previously been described as being immunodominant for SARS-CoV (Wang et al., 2016). PE (antigen containing highly conserved epitopes for SARS-CoV and SARS-CoV-2) reacted only with Abs to the full-size S protein of SARS-CoV. Nevertheless, PE was also selected for vaccine development. Conserved S2 subunit epitopes could be a potential target for therapy and vaccine design (Shah et al., 2021).

The current research proposed and characterized a vaccine candidate that represents the compositions of three CoV recombinant antigens and spherical particles (SPs) generated by the thermal remodeling of TMV (SPs + 3AG). SPs served as an adjuvant. The immunogenicity of the vaccine candidate (SPs + 3AG: 250 μg of SPs + 7 μg of each antigen) was evaluated and compared with the immunogenicity of 3AG mixture without SPs. Abs titers to Co1, CoF, and PE were assessed 14 days after the second immunization of mice. The immune response to the Co1 antigen (corresponding to the RBD) was shown to be quite low in both cases: with SPs or without. The low immunogenicity of the RBD-based subunit vaccines derived from insect and mammalian expression systems has been reported previously (Yang et al., 2020; Tan et al., 2021). In particular, Yang et al. (2020) presented data on low titers of Abs to RBD in the serum of mice immuniszed twice with 5 μg of RBD in the presence of aluminum hydroxide adjuvant. However, RBD-specific Abs titers increased after the second vaccine boost (Yang et al., 2020). Some recent studies have shown that the efficient production of SARS-CoV-2 RBD in *E. coli* may imply the refolding (with buffer containing L-arginine, glutathione, glutathione disulfide, urea) or co-expression of sulfhydryl oxidase and disulfide isomerase to rebuild the disulfide bonds of the target protein (He et al., 2021; Prahlad et al., 2021). The results of the current research have demonstrated the low immunogenicity of recombinant RBD and the fact that two immunizations are insufficient to provide the required RBD-specific Abs titers, even in the case of immunization with adjuvant. At the same time, the immunogenicity of CoF (constituting RBD fused to the conserved 29 aa-length epitope of the S2 subunit), as well as PE (containing the conserved fragment including HR1 and HR2 of the S2 subunit) within SPs + 3AG compositions, was high. SPs significantly enhanced the immune response to CoF and PE compared to the immune response induced by the 3AG. Moreover, in the present study, SPs-provided enhancement of CoF and PE immunogenicity was demonstrated not only for total IgG titers, but also for IgG1, IgG2a, IgG2b, and IgG3 isotypes separately. This is an indirect indication that the vaccine candidate stimulated both a Th-1-mediated, and a Th-2-mediated, immune response. IgG2a/IgG1 ratio for PE antigen was 1 and for CoF antigen was 2.4. Therefore, SPs provide a well-balanced activation of an immune response and could be an effective adjuvant for CoV antigens. The data presented on CoF and PE immunogenicity are in accordance with a recent study in which nanoparticles containing RBD and the conserved fragment of the S2 subunit (HR) had been used for vaccine development (Ma et al., 2020). It was revealed that ferritin nanoparticles conjugated to RBD and HR (RBD-HR nanoparticles) and mixed with a Sigma Adjuvant System (an alternative to Freund’s Adjuvant) elicited both humoral and cellular responses after two immunizations of mice. RBD-HR nanoparticles, as well as RBD nanoparticles (RBD conjugated with ferritin nanoparticles), had protected human ACE2 (hACE2) mice against SARS-CoV-2 infection, but only RBD-HR nanoparticles had been shown to cause the formation of nAbs, not only against SARS-CoV-2, but also against other coronaviruses (Ma et al., 2020). Therefore, it can be suggested that Abs induced by CoF and PE containing conserved S2 subunit fragments could play a crucial role in protection against a broad spectrum of SARS-related coronaviruses.

As SPs protein is antigenically alien to mammals, the production of anti-SPs Abs can be expected. Previously, we found that immunizations with other SPs-antigen compositions induced lower Abs titers to SPs than Abs titers to model antigens (Trifonova et al., 2017; Evtushenko et al., 2020). Here, SPs immunogenicity in SPs + 3AG compositions was confirmed to be low. Total IgG titers to SPs were nine times and 16 times lower than total IgG titers to CoF and PE, respectively. Thus, high antigen-specific Abs titers simultaneously with low adjuvant-specific Abs titers is a unique property of SPs. As shown previously, adjuvant-specific Abs titers were higher than, or approximately equal to, Abs titers against antigens in the case of other protein-based adjuvants (Koo et al., 1999; Kalnciema et al., 2012; Rioux et al., 2012). For example, the aforementioned CoV vaccine from ferritin-conjugated RBD-HR chimeric nanoparticles stimulated in excess of ten times more Abs titers to ferritin than to CoV antigens. The authors supposed that *Helicobacter pylori* ferritin and Abs against it should not be toxic, but this problem nevertheless remains under discussion (Ma et al., 2020). It can be assumed that high CoV-specific Abs titers and low Abs titers against the adjuvant (SPs) is an encouraging indicator of SPs’ efficacy.

On another stage of the current proof-of-concept study, sera neutralizing activity from ten hamsters immunized with SPs + 3AG compositions was tested. Syrian hamsters are considered to be an acceptable model for studying vaccines against COVID-19 (Imai et al., 2020). The NT50 (the serum dilution needed to inhibit 50% virus replication) ranged from 17.82 to 86.92. The SARS-CoV-2 neutralization assay is a crucial method for assessing antibody-mediated protection in infected and vaccinated people. However, it is difficult to compare results from different studies. Laboratories use various approaches to evaluating nAbs titers: live virus neutralization assay, pseudovirus neutralization assay, etc. (Lu et al., 2021). There are also no standard metrics for presenting the results of neutralization analysis (Gundlapalli et al., 2020). It also should be noted that nAbs levels do not always correlate with the protective efficacy of the vaccine candidate (Gao et al., 2020; Yang et al., 2020).

Here, SPs + 3AG compositions have been suggested as a CoV vaccine candidate. SPs (spherical particles derived from TMV) have been shown to significantly increase total IgG antibody titers to coronavirus antigens within vaccine candidate compositions, so their suitability for being applied as an adjuvant has been proven. Since the set of antigens developed includes epitopes being conserved for a number of betacoronaviruses, SPs + 3AG compositions could be preventative not only for SARS-CoV-2, but also for SARS-CoV and other potentially dangerous SARS-related coronaviruses. However, the heterologous protection and its durability need further research, which will be conducted along with SPs + 3AG compositions detailed characterization being required for clinical study initiation. The successful expression of the antigens in *E. coli* demonstrated in the current work is a scalable, efficient and cost-effective approach to protein production. The ability to induce nAbs against SARS-CoV-2 revealed in immunized hamsters makes SPs + 3AG a highly promising candidate for ongoing preclinical studies.

## Data Availability Statement

The original contributions presented in the study are included in the article/Supplementary Material, further inquiries can be directed to the corresponding author/s.

## Ethics Statement

The animal study was reviewed and approved by the ethics committee of the Lomonosov Moscow State University (Application #136-a, version 3, approved during the Bioethics Commission meeting #134-d held on 19.08.2021) (for study on mice) and by the Commission on Bioethics (BEC) of a Group of Scientific Research Institutes (protocol #9.73/20 dated 28.12.2020) (for study on hamsters).

## Author Contributions

OVK conceptualized and supervised the study. AK, ER, EE, OK, PI and NN were involved in the design of the study. AK, ER, EE, TM, and MA carried out the experiments. VG performed and analyzed the virus neutralization assay. AK, ER, and EE contributed to data analysis and interpretation. All authors contributed to editing of the manuscript.

## Conflict of Interest

The authors declare that the research was conducted in the absence of any commercial or financial relationships that could be construed as a potential conflict of interest.

## Publisher’s Note

All claims expressed in this article are solely those of the authors and do not necessarily represent those of their affiliated organizations, or those of the publisher, the editors and the reviewers. Any product that may be evaluated in this article, or claim that may be made by its manufacturer, is not guaranteed or endorsed by the publisher.

## Footnotes

1
http://expasy.org

2
https://www.who.int/publications/m/item/draft-landscape-of-covid-19-candidate-vaccines

## Supplementary Material

Supplementary Figure 1 Characterization of SPs. Transmission electron microscopy, without staining. Scale bar, 2 μm.

Supplementary Figure 2 Negative controls for immunofluorescent analysis (SPs without 3AG). **(A)** and **(B)**, **(C)** and **(D)** In pairs are the same images presented in fluorescence and phase contrast modes, respectively. The SPs were treated with polyclonal anti-spike (SARS-CoV) Abs **(A,B)** or polyclonal anti-spike (SARS-CoV-2) Abs **(C,D)** and secondary Abs conjugated to Alexa Fluor ^®^ 546. Scale bars, 5 μm. Complexes were obtained in 1 x PBS. Abs—antibodies.
