# Supplementary figures and images for: Vaccine Candidate Against COVID-19 Based on Structurally Modified Plant Virus as an Adjuvant
Source: Front Microbiol. 2022 Feb 28;13:845316. doi: 10.3389/fmicb.2022.845316 (PMC8919459; doi:10.3389/fmicb.2022.845316)

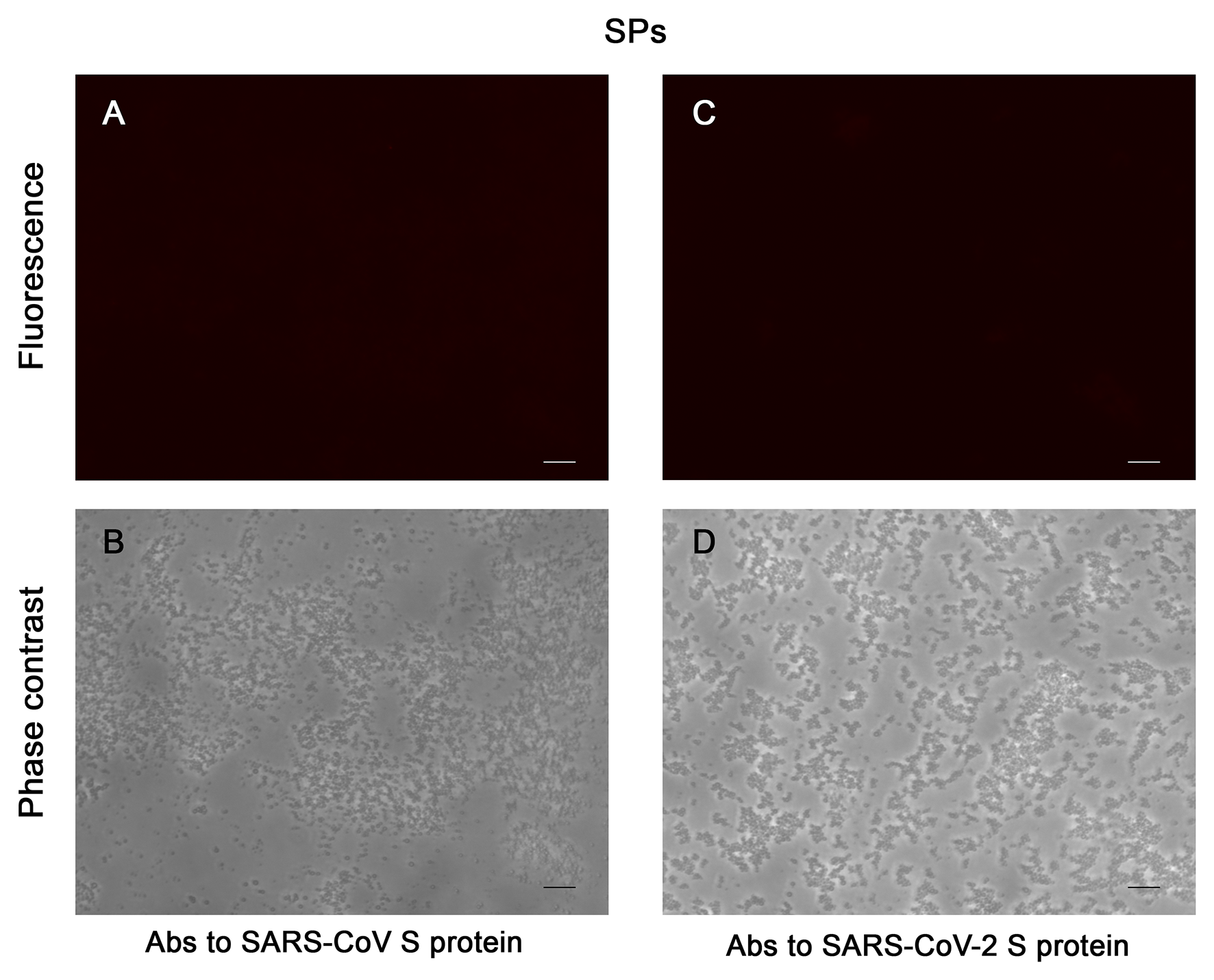

Supplement: Supplementary Figure 2 — Negative controls for immunofluorescent analysis (SPs without 3AG). (A) and (B), (C) and (D) In pairs are the same images presented in fluorescence and phase contrast modes, respectively. The SPs were treated with polyclonal anti-spike (SARS-CoV) Abs (A,B) or polyclonal anti-spike (SARS-CoV-2) Abs (C,D) and secondary Abs conjugated to Alexa Fluor ® 546. Scale bars, 5 μm. Complexes were obtained in 1 x PBS. Abs—antibodies. [file Image_2.TIF]
