# Supplementary material for: Vaccine Candidate Against COVID-19 Based on Structurally Modified Plant Virus as an Adjuvant
Source: Front Microbiol. 2022 Feb 28;13:845316. doi: 10.3389/fmicb.2022.845316 (PMC8919459; doi:10.3389/fmicb.2022.845316)
Supplement: Supplementary file 6 [file Table_4.DOCX]

| **Titers of mice sera titrated on SPs** | | |
| --- | --- | --- |
| group | identification  number of mouse | titer |
|  |  | IgG total |
| group 1 (PBS) | **Median** | **1318** |
|  | 1 | 786 |
|  | 2 | 1318 |
|  | 3 | 1107 |
|  | 4 | 3157 |
|  | 5 | 2342 |
| group 3 (SPs + coronavirus recombinant antigens) | **Median** | **3168** |
|  | 16 | 3064 |
|  | 17 | 15453 |
|  | 18 | 4870 |
|  | 19 | 802 |
|  | 20 | 1275 |
|  | 21 | 1574 |
|  | 22 | 3272 |
|  | 23 | 8391 |
|  | 24 | 2594 |
|  | 25 | 7438 |

**Supplementary Table 4.** Titers of mice sera titrated on SPs. Group of mice (group 3) was immunised intraperitoneally twice (days 0, 15), with 21 μg of coronavirus recombinant antigens in compositions with SPs (250 µg). The control group was immunised with PBS. All administered samples were in PBS in a total volume of 0.2 ml. Blood was collected after the second immunisation on the 29^th^ day of the experiment. Sera titers were evaluated using an indirect ELISA with anti-mouse HRP conjugate against IgG (ab6728). Concentration of SPs on microplate – 10 μg/ml.
