# Supplementary material for: Vaccine Candidate Against COVID-19 Based on Structurally Modified Plant Virus as an Adjuvant
Source: Front Microbiol. 2022 Feb 28;13:845316. doi: 10.3389/fmicb.2022.845316 (PMC8919459; doi:10.3389/fmicb.2022.845316)
Supplement: Supplementary file 7 [file Table_5.DOCX]

| **Body weight of animals during the immunisation period** | | | |
| --- | --- | --- | --- |
| identification number of hamster | day 0 | day 21 | day 42* |
| 1 | 103 | 105 | 100 |
| 2 | 98 | 104 | 99 |
| 3 | 81 | 95 | 98 |
| 4 | 97 | 109 | 104 |
| 5 | 106 | 117 | 109 |
| 6 | 104 | 106 | 116 |
| 7 | 96 | 112 | 110 |
| 8 | 64 | 73 | 78 |
| 9 | 95 | 108 | 109 |
| 10 | 96 | 110 | 109 |

**Supplementary Table 5**. Hamsters body weight during the immunisation period (grammes).

* - weight was analysed a day after the blood collection.
